# TATA-Binding Protein-Based Virtual Screening of FDA Drugs Identified New Anti-Giardiasis Agents

**DOI:** 10.3390/ijms25116238

**Published:** 2024-06-05

**Authors:** Carlos Gaona-López, Domingo Méndez-Álvarez, Adriana Moreno-Rodríguez, Juan Luis Bautista-Martínez, José Antonio De Fuentes-Vicente, Benjamín Nogueda-Torres, Itzhel García-Torres, Gabriel López-Velázquez, Gildardo Rivera

**Affiliations:** 1 Laboratorio de Biotecnología Farmacéutica, Centro de Biotecnología Genómica, Instituto Politécnico Nacional, Reynosa 88710, Mexico; doomadv@hotmail.com; 2 Laboratorio de Estudios Epidemiológicos, Clínicos, Diseños Experimentales e Investigación, Facultad de Ciencias Químicas, Universidad Autónoma “Benito Juárez” de Oaxaca, Oaxaca 68120, Mexico; arimor10@hotmail.com (A.M.-R.); jlbautistam@gmail.com (J.L.B.-M.); 3 Instituto de Ciencias Biológicas, Universidad de Ciencias y Artes de Chiapas, Tuxtla Gutiérrez 29039, Mexico; jose.defuentes@unicach.mx; 4 Departamento de Parasitología, Escuela Nacional de Ciencias Biológicas, Instituto Politécnico Nacional, Ciudad de México 11340, Mexico; bnogueda@ipn.mx; 5 Laboratorio de Biomoléculas y Salud Infantil, Instituto Nacional de Pediatría, Ciudad de México 04530, Mexico; garciaitzhel@gmail.com (I.G.-T.); glv_1999@ciencias.unam.mx (G.L.-V.)

**Keywords:** *Giardia lamblia*, drug repositioning, FDA, inhibitors, TATA-binding protein

## Abstract

Parasitic diseases, predominantly prevalent in developing countries, are increasingly spreading to high-income nations due to shifting migration patterns. The World Health Organization (WHO) estimates approximately 300 million annual cases of giardiasis. The emergence of drug resistance and associated side effects necessitates urgent research to address this growing health concern. In this study, we evaluated over eleven thousand pharmacological compounds sourced from the FDA database to assess their impact on the TATA-binding protein (TBP) of the early diverging protist *Giardia lamblia*, which holds medical significance. We identified a selection of potential pharmacological compounds for combating this parasitic disease through in silico analysis, employing molecular modeling techniques such as homology modeling, molecular docking, and molecular dynamics simulations. Notably, our findings highlight compounds DB07352 and DB08399 as promising candidates for inhibiting the TBP of *Giardia lamblia.* Also, these compounds and DB15584 demonstrated high efficacy against trophozoites in vitro. In summary, this study identifies compounds with the potential to combat giardiasis, offering the prospect of specific therapies and providing a robust foundation for future research.

## 1. Introduction

Parasitic diseases caused by protists represent a significant burden for the health systems of the different countries where they are endemic [1,2]. Similarly, ongoing migratory flows, influenced by various factors, have disseminated these diseases to regions where they are not typically prevalent [3]. The parasitosis caused by the protists *Giardia lamblia* (*G. lamblia*) represents a significant economic burden, especially for Latin American countries [4]. The disease produced by this parasite can be lethal or cause morbidity; despite the availability of various drugs for treating this disease, it is crucial to highlight that some of them have adverse effects ranging from mild to severe [5]. Additionally, there have been reports of an elevated occurrence of resistance to drugs commonly utilized to combat this illness [6]. Delayed treatment of this parasitosis and a weakened immune system render this disease a significant source of mortality and morbidity, particularly in the most impoverished regions [4,5].

Giardiasis is a parasitic disease with a global distribution, estimated to cause more than 300 million cases annually [7]. The most vulnerable group affected by this disease is children, for whom it can be lethal [8]. In 2004, this parasitic infection was included in the *Neglected Diseases* initiative by the World Health Organization (WHO) [9]. *G. lamblia* has a simple life cycle, which consists of two stages: the trophozoite, which lives in the duodenum (the anterior part of the small intestine), where, with the help of its ventral disc, it attaches to the epithelium of the intestine; and the cyst, which is the infective form and is excreted through the feces of infected individuals [10]. This cyst can remain dormant for months at warm temperatures [10]. The main drugs to combat giardiasis are those 5-nitroimidazole derivatives (metronidazole, tinidazole, secnidazole, and ornidazole), which have strong reductive activity against facultative anaerobic parasites such as *G. lamblia*, where damage to the parasite’s DNA occurs [11,12].

Since numerous medications employed for treating this parasite originated in the mid-20th century, there is now an escalation in drug resistance. Additionally, the noted adverse effects underscore the significance of creating new drugs and investigating fresh therapeutic targets to successfully address these diseases, impacting millions worldwide.

Recently, transcription factors have been considered a promising target for treating parasitic diseases. This is mainly attributed to the differences in protein sequences regarding higher eukaryotes and the reported low mutation rate [13,14,15,16,17,18,19].

The TATA-binding protein (TBP) is a universal transcription factor, considered ubiquitous and essential for the initiation process of the three transcription systems reported in eukaryotes (RNApol1, RNApol2, and RNApol3) [20]. Considering the significant variation observed in the amino acid sequences of TBPs from early branching protists [13,14,15,16], like the one studied here, and the low mutation rate found in this protein and other transcription factors [17,18], we suggest TBP as a promising candidate for targeted therapy against giardiasis since multiple crystallographic studies have determined that the entire surface of the TBP interacts with other transcription factors [15]. Thus, altering these interactions by occluding the binding site between proteins could prevent the pre-initiation complex assembly, thereby suppressing the transcription of the three RNA polymerases. An example of this has been reported for the experimental cancer treatment drug, CX-5461, which inhibits the interaction between RNApol1 and the SL1/TBP complex, preventing pre-initiation complex formation [21]. Additionally, inhibiting interactions with other transcription factors using small molecules could be lethal for the parasite. Negative Cofactor 2 (NC2) is a negative TBP regulator, which prevents TFIIA and TFIIB binding (critical components in the transcription initiation complex) and is responsible for stabilizing RNA polymerase. A molecule with the necessary affinity for the NC2 motif could similarly hinder the formation of the preinitiation complex by occluding TFIIA and TFIIB, to the detriment of the parasite. These types of inhibition strategies are further explained by Lambert et al., 2018 and Walters et al., 2021, with perspectives for treating cancer and parasitic diseases caused by protists [19,22].

For this reason, we aimed to assess over eleven thousand pharmacological compounds sourced from the FDA database, evaluate their potential, and explore the possibility of repurposing them to treat this parasitic disease. In this study, we employed molecular modeling techniques such as homology modeling, molecular docking, molecular dynamics, and MM-PBSA analysis (a computational technique used in molecular dynamics simulations to estimate the free energy of binding between a ligand and a receptor) to evaluate the inhibitory response of 7969 compounds sourced from the FDA database against the TBP of *G. lamblia* (*Gl*TBP). To accomplish this, we analyzed the interactions between pharmacological compounds that passed the filter based on Lipinski’s Rule of Five and the NC2 motif of TBP of *G. lamblia*, along with their impact on the human TBP (*Hs*TBP). Then, we selected those compounds with the highest selectivity for the TPB of *G. lamblia* regarding *Hs*TBP. Finally, three of the best-ranked compounds were assayed in vitro against *G. lamblia* trophozoites.

## 2. Results

### 2.1. Multiple Sequence Alignments

Due to the absence of experimentally resolved three-dimensional structures for the *Gl*TBP, we resorted to recovering the crystallographic structures of different eukaryotic species belonging to several taxonomic groups, including animals, plants, and fungi. This was carried out with the purpose of conducting a structural alignment that would serve as a reference for sequences whose structures were not resolved. The crystallographic structures (PDB code: 1JFI, 1QNA, 1NH2, and 3OCI) were used to identify the DNA binding domain in the TBP of the parasite under investigation.

**Figure 1:**
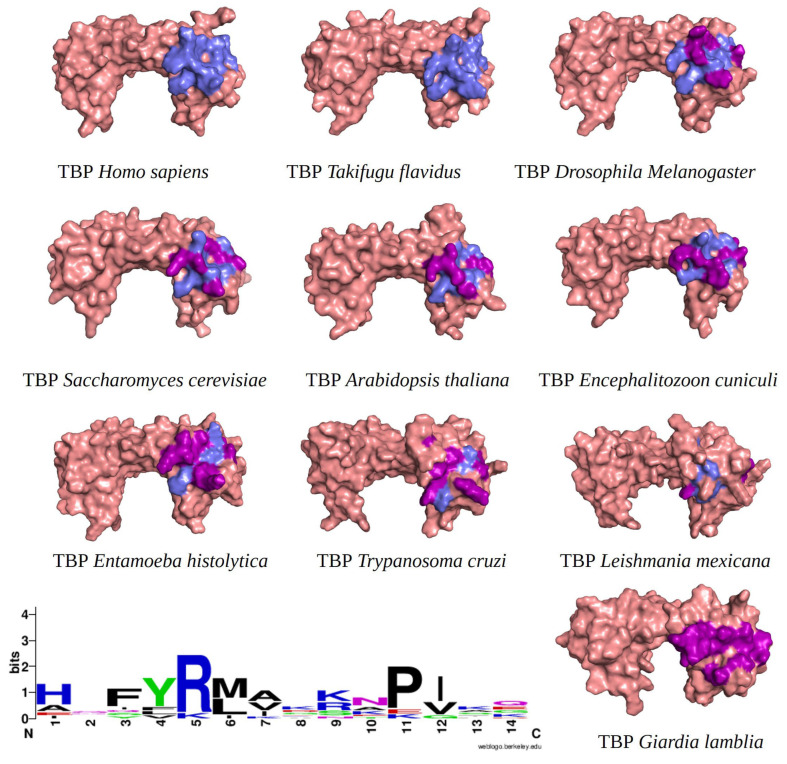
TBPs eukaryotic with the 14 amino acid residues involved in the formation of the NC2 motif highlighted. Residues conserved with regarding *Hs*TBP are shown in slate color, while non-conserved residues are shown in purple. In the bottom left corner of the image, the WebLogo diagram that outlines the conservation of the 14 amino acid residues that make up the NC2 motif.

Furthermore, we incorporated sequences of TBPs from different taxonomic groups of eukaryotes into our alignment. This allowed us to evaluate the conservation of amino acids comprising the NC2 motif. We emphasize that a lower conservation of amino acids on the binding site indicates a higher likelihood of molecules with pharmacological potential capable of interfering with the proper assembly of the transcription machinery. As aforementioned, the structural alignment served as a foundation for aligning the remaining sequences with unresolved three-dimensional structures. This is based on the premise that a protein’s three-dimensional structure tends to be more conserved throughout evolution than its primary amino acid sequence. This is especially true for ancient protein families, whose proteins share a similar function and structure due to their common evolutionary ancestry [23,24]. Furthermore, the amino acid residues known to interact with NC2 are highlighted in the different three-dimensional models (Figure 1).

It is worth noting that the amino acid residues involved in the interaction with NC2 are fully conserved in vertebrates, as previously reported for TBPs in general within this group of animals [18]. However, as the evolutionary distance increases, the number of residues changed also increases. For instance, in *Arabidopsis thaliana* (*A. thaliana)*, *Encephalitozoon cuniculi* (*E. cuniculi)*, and *Saccharomyces cerevisiae (S. cerevisiae)*, 7 out of the 14 residues involved in the interaction with NC2 are conserved. Notably, in *Entamoeba histolytica* (*E. histolytica)*, *Leishmania mexicana* (*L. mexicana*), and *Trypanosoma cruzi* (*T. cruzi)*, only 3 out of the 14 conserved residues are retained. In *G. lamblia*, all 14 residues responsible for interacting with NC2 have been altered (Figure 1).

### 2.2. Docking Molecular Analysis 

**Figure 2:**
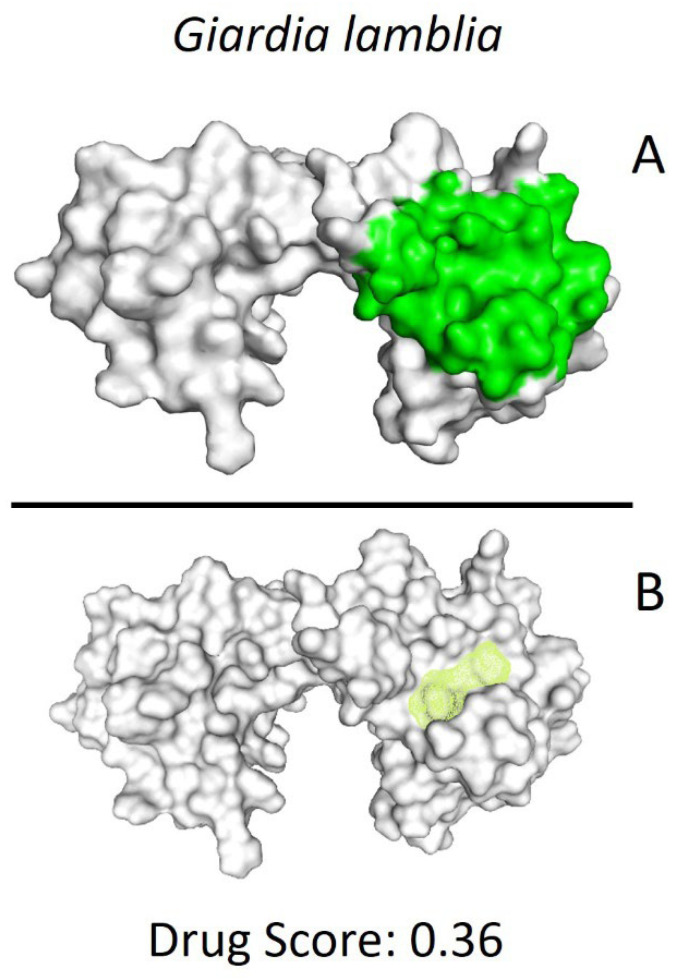
Potential ligand binding sites. (**A**) Results of blind docking, (**B**) Potential druggable sites, according to the DoGSiteScorer Binding Site server. For *G. lamblia*, the second-best druggable site is indicated, as denoted by its Drug Score. The NC2 site is also reported in the literature as a potential therapeutic target in parasites, including protozoa such as *Plasmodium falciparum* (*P. falciparum*) and *E. histolytica*.

After identifying the potential binding site (Figure 2), molecular modeling techniques were employed to predict the interactions of the compounds previously screened in the Drug Bank database. This process helped identify compounds with the highest affinity and selectivity for the binding site of the *Gl*TBP.

**Figure 3:**
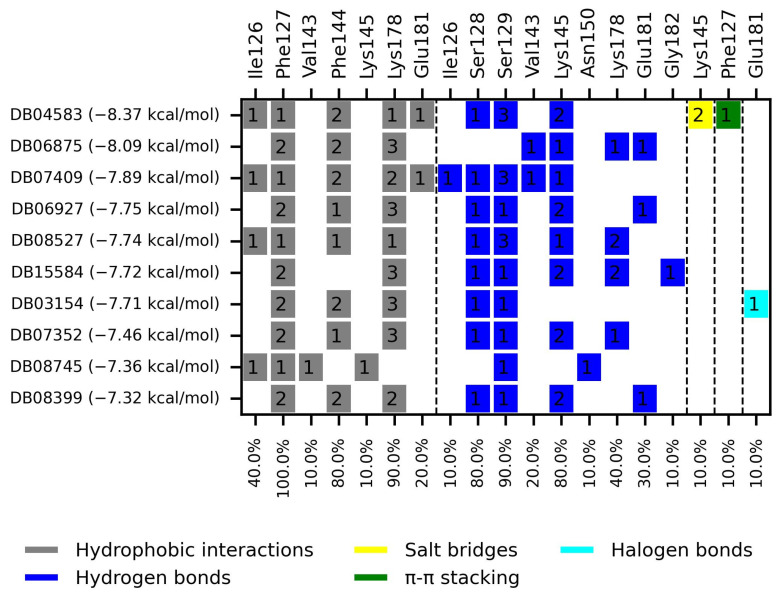
Interaction chart of the top ten compounds with better selectivity for *Gl*TBP compared to *Hs*TBP. On the x-axis, the percentage of interactions of each amino acid residue with the ligands is shown, while on the y-axis, the interacting compound is displayed along with its BFE value. The numbers within each box indicate the number of bonds formed by each amino acid residue.

**Figure 4:**
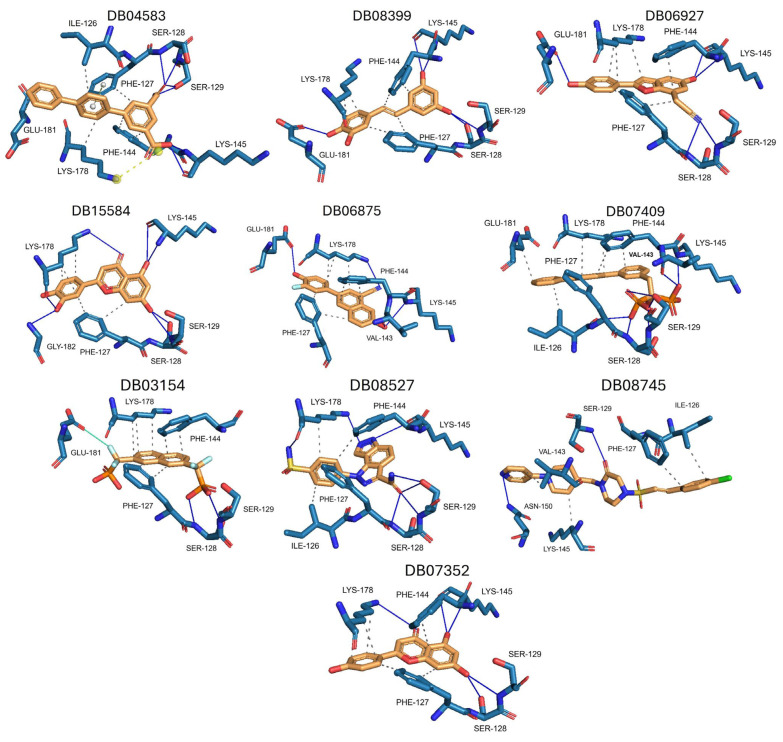
Interactions profile between amino acid residues of the NC2 motif and various ligands. The ten compounds with the highest selectivity index for *Gl*TBP in comparison to *Hs*TBP are displayed. Dark blue lines represent hydrogen bonds, black dashes represent hydrophobic-type interactions, light blue lines represent halogen-type bonds, grey lines represent π-stacking, and yellow dashes represent salt bridges.

The ten compounds with the highest selectivity for *Gl*TBP over *Hs*TBP were DB03154, DB04583, DB06875, DB06927, DB07352, DB07409, DB08399, DB08527, DB08745, and DB15584 (Table 1). This table shows the structure of compounds in 2D, the pharmacological target, the giardicidal effect reported, the binding free energy (BFE) values, the interaction profile found, and other reported functions. Additionally, Figure 3 shows an interaction chart, and Figure 4 shows interactions between ligands and the NC2 motif.

DB04583 had a BFE value of −8.365 kcal/mol, with a selectivity of −2.2 with respect to *Hs*TBP. This compound showed five hydrophobic-type interactions with the amino acid residues Ile126, Phe127, Phe144, Lys178, and Glu181. Additionally, three hydrogen bonds are shown with residues Ser128, Ser129, and Lys145. A salt bridge with Lys178 and a π-stacking interaction was identified with Phe127.

DB08399 had a BFE value of −7.318 kcal/mol, with a selectivity of −2.06 with respect to *Hs*TBP. This compound showed four hydrophobic-type interactions with the amino acid residues Phe127, Phe144, and Lys178. Additionally, four hydrogen bonds are shown with residues Ser128, Ser129, Lys145, and Glu181. It is worth mentioning that there is a precedent for this compound with anti-Giardia activity, demonstrating an effect on the assembly of microtubules [26].

DB06927 had a BFE value of −7.749 kcal/mol, with a selectivity of −2.04 with respect to *Hs*TBP. This compound showed three hydrophobic-type interactions with the amino acid residues Phe127, Phe144, and Lys178. Additionally, four hydrogen bonds are shown with residues Ser128, Ser129, Lys145, and Glu181. DB06927’s human target is the nuclear receptor coactivator 1 (NCOA1). This suggests this compound’s potential presence in the parasite’s nucleus [40].

DB15584 (luteolin) had a BFE value of −7.720 kcal/mol, with a selectivity of −1.92 with respect to *Hs*TBP. This compound showed two hydrophobic-type interactions with the amino acid residues Phe127, and Lys178. Additionally, five hydrogen bonds are shown with residues Ser128, Ser129, Lys145, Lys178, and Gly182. Studies conducted by Palomo-Ligas et al. in 2022 highlighted the presence of various compounds in pomegranate peel extract, including ellagitannins, flavones (such as luteolin, DB15584), and ellagic acid. These authors mention that this extract inhibits the growth and decreases the adhesion of trophozoites by 74.36% and 46.8%, respectively, in trophozoites treated with 200 μg/mL. They also report an IC_50_ of 179 μg/mL, highlighting alterations in the tubulin gene’s expression and the distribution of this protein within the cell [28].

DB06875 (ERB-196) had a BFE value of −8.090 kcal/mol, with a selectivity of −1.88 with respect to *Hs*TBP. This compound showed three hydrophobic-type interactions with the amino acid residues Phe127, Phe144, and Lys178. Additionally, four hydrogen bonds are shown with residues Val143, Lys145, Lys178, and Glu181. DB06875’s human target is the nuclear receptor coactivator 1 (NCOA1). This suggests this compound’s potential presence in the parasite’s nucleus [40].

DB07409 had a BFE value of −7.888 kcal/mol, with a selectivity of −1.84 with respect to *Hs*TBP. This compound showed five hydrophobic-type interactions with the amino acid residues Ile126, Phe127, Phe144, Lys178, and Glu181. Additionally, four hydrogen bonds are shown with residues Ile126, Ser128, Ser129, Val143, and Lys145. 

DB03154 had a BFE of −7.712 kcal/mol, with a selectivity of −1.84 with respect to *Hs*TBP. This compound showed three hydrophobic-type interactions with the amino acid residues Phe127, Phe144, and Lys178. Additionally, two hydrogen bonds are shown with residues Ser128 and Ser129. Finally, a halogen-type bond (HalB) is present with Glu181.

DB08527 had a BFE value of −7.743 kcal/mol, with a selectivity −1.82 with respect to *Hs*TBP. This compound showed four hydrophobic-type interactions with the amino acid residues Ile126, Phe127, Phe144, and Lys178. Additionally, four hydrogen bonds are shown with residues Ser128, Ser129, Lys145, and Lys178.

DB08745 had a BFE of −7.355 kcal/mol, with a selectivity of −1.82 with respect to *Hs*TBP. This compound showed four hydrophobic-type interactions with the amino acid residues Ile126, Phe127, Val143, and Lys145. Additionally, two hydrogen bonds are shown with residues Ser129 and Asn150.

DB07352 (Apigenin) had a BFE of −7.459 kcal/mol, with a selectivity of −1.806 with respect to *Hs*TBP. This compound showed three hydrophobic-type interactions with the amino acid residues Phe127, Phe144, and Lys178. Additionally, four hydrogen bonds are shown with residues Ser128, Ser129, Lys145, and Lys178. It is pertinent to mention that Davoodi et al., 2018, reported an anti-giardia activity of the hydroalcoholic extract from *Origanum vulgare*, demonstrating a giardicidal effect at concentrations of 200 mg/kg, similar to metronidazole. The authors propose that the anti-giardia activity is due to the presence of phenolic compounds and flavonoids, among which is DB07352 (Apigenin) [34].

### 2.3. Molecular Dynamics Simulation

For our molecular dynamics simulation, we selected compounds that were economically accessible and competitive (molport.com, accessed on 5 October 2023) with currently available drugs, considering that giardiasis primarily affects impoverished regions. The compounds evaluated through molecular dynamics simulation (by triplicate) were DB08399 (Top 2), DB15584 (Top 4), and DB07352 (Top 10) for *G. lamblia*.

**Figure 5:**
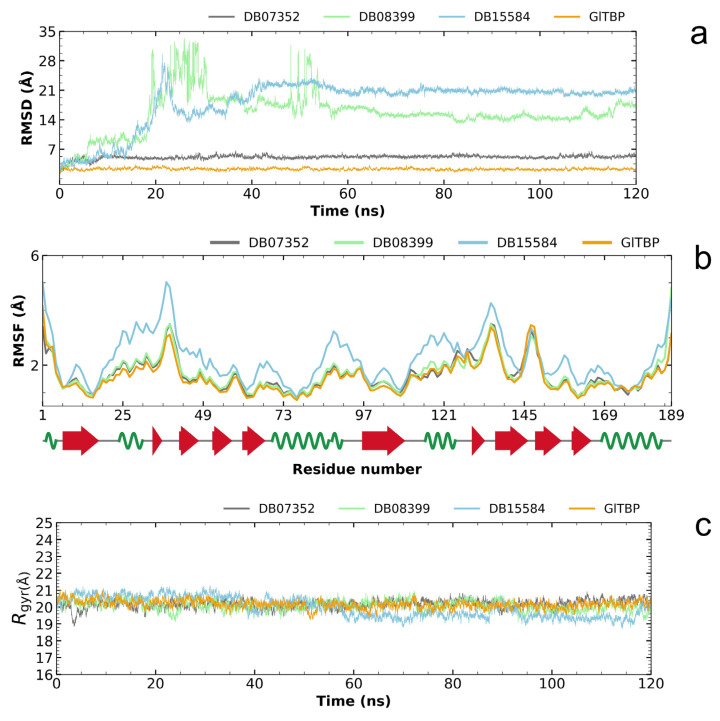
Illustration of the evaluation of the RMSD plot (**a**), RMSF plot (**b**), and radius of gyration plot (**c**) for *Gl*TBP in both its isolated states and when in complex with potential inhibitors.

Analysis of the RMSD plot enabled us to identify compounds that exhibit stability within the designated binding site, corresponding to the NC2 motif. Regarding *Gl*TBP, the two best compounds with values that showed acceptable differences between the maximum and minimum fluctuations were the compounds DB07352 and DB08399, which exhibit maximum and minimum average fluctuation differences of 5.10 ± 0.49 Å and 15.63 ± 4.83 Å, respectively. Notably, DB07352 achieves stabilization within the few initial nanoseconds of the simulation and sustains this stability throughout the remainder of the simulated trajectory (Figure 5a).

Conversely, the complex formed by *Gl*TBP and compound DB15584 exhibits significant fluctuation throughout the simulation, which has led to its exclusion from future in vitro testing against *Gl*TBP. It is noteworthy that this compound is a component of pomegranate peel extract. It has been confirmed that this extract possesses inhibitory properties that affect the growth of *G. lamblia* trophozoites and, in addition, diminish the parasite’s ability to adhere to the inner wall of the duodenum. Furthermore, it is important to note that the authors report modifications in tubulin gene expression and its intracellular localization [28].

By analyzing the RMSF graph, we assess the disturbances observed in *Gl*TBP, both when they are isolated and when they are in complex with different compounds. In the instance of *Gl*TBP, the complexes involving DB07352 and DB08399 exhibit RMSF profiles similar to that of the protein in its unbound state. This suggests that the binding of these compounds does not induce significant disruptions in the protein’s structure, pointing out the remarkable stability of *Gl*TBP. 

Conversely, it is noteworthy to emphasize the complex formed between *Gl*TBP and DB15584, which displays a distinct RMSF profile compared to the other complexes (refer to Figure 5b). Owing to this disparity, we have opted to exclude compound DB15584 from any further in vitro assessments against *Gl*TBP.

Finally, based on the analysis of the radius of gyration, it can be inferred that *Gl*TBP-free remained stable throughout the molecular dynamics simulation (see Figure 5c). Differences in fluctuations between the maximum and minimum values are observed, with an average value of 20.17 ± 0.24 Å for this protein.

In the case of *Gl*TBP in complex with its ligands, the complexes exhibit differences in fluctuations on the order of 20.25 ± 0.28 Å and 20.10 ± 0.30 Å to DB07352 and DB08399, respectively. However, the case of compound DB15584 in complex with *Gl*TBP is worth noting, which shows a difference of 19.97 ± 0.59 Å (see Figure 5c). 

For reference, the radius of gyration of *Hs*TBP (PDB ID 1JFI) in its isolated state exhibits a fluctuation difference of 2.1911 angstroms.

### 2.4. Molecular Dynamics Analysis of the Two Compounds with Potential for Repositioning in GlTBP and Their Evaluation on HsTBP

**Figure 6:**
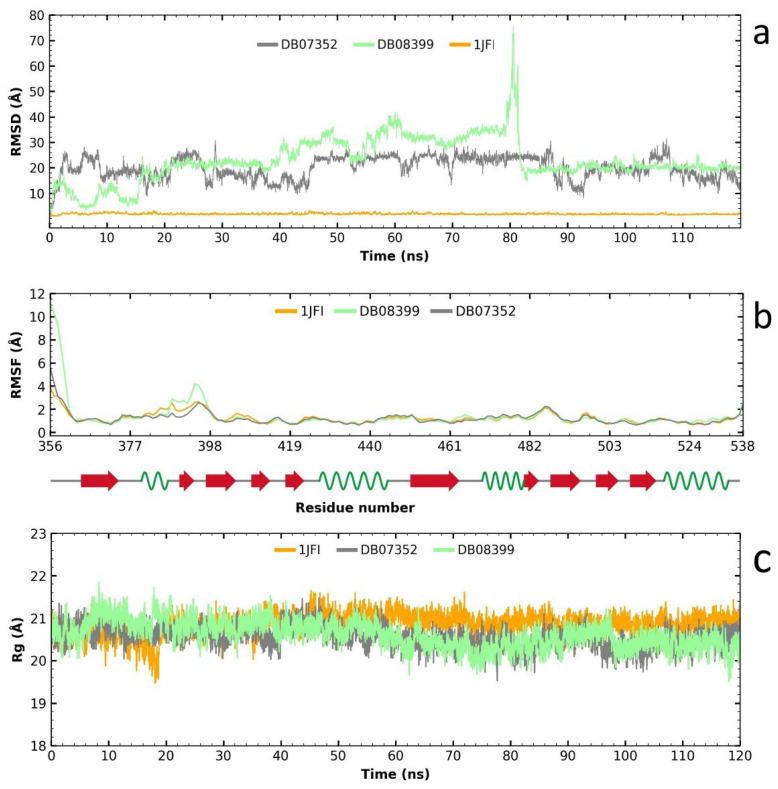
Analysis of RMSD, RMSF, and gyration radius for the two potential inhibitors of the TATA-binding protein (TBP) in *G. lamblia* and their impact on *Hs*TBP. The analysis shows high fluctuations for two compounds with *Hs*TBP, indicating high specificity for *Gl*TBP (**a**). RMSF analysis shows minimal fluctuation except for DB08399, especially between residues 387 and 398 (**b**). The protein remains stable throughout (**c**). Thus, high RMSD values are due to the reduced stability of the compounds in *Hs*TBP, favoring higher selectivity for *Gl*TBP.

Following the RMSD analysis, we observe that the two compounds exhibit very high fluctuations in their complex with *Hs*TBP (Figure 6a), which suggests a high specificity for *Gl*TBP. Analyzing the RMSF graph reveals minimal fluctuation along the protein structure, except for the complex formed with compound DB08399, which shows greater fluctuation than the other complexes and the protein alone, particularly between amino acid residues 387 and 398 (Figure 6b). Finally, the gyration radius graph indicates that the protein, both alone and in the complex, remains stable throughout the simulation. Therefore, the elevated values observed in the RMSD graph are attributed to the reduced stability of the two compounds in *Hs*TBP, favoring a higher inhibitory selectivity for *Gl*TBP.

In summary, these findings support the hypothesis that the evaluated compounds DB07352 and DB08399 could have effective potential for repositioning against *Gl*TBP, with promising implications for the development of selective inhibitors.

### 2.5. MM-PBSA Analysis

The MMPBSA analysis revealed the molecular interactions present in the two protein–ligand complexes that exhibited the best RMSD values in *Gl*TBP, providing insights into the factors contributing to the binding affinity in these complexes. In *Gl*TBP, ∆Gb values of −19.28 and −14.16 kcal/mol are shown for the complexes formed with DB07352 and DB08399, respectively (Table 2).

**Figure 7:**
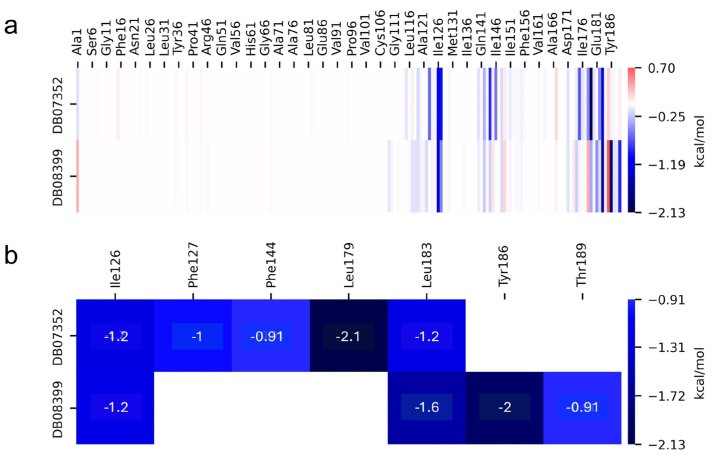
Relative contribution of different residues to the free binding energy with *Gl*TBP. Residues showing some interaction with DB07352 and DB08399 (**a**). Key residues contributing energy to ligand binding (**b**).

On the other hand, the individual contributions of different residues from *Gl*TBP to the binding energy were obtained for the last 40 ns of the molecular dynamics analysis, aiming to identify key residues in the protein–ligand interaction. In *Gl*TBP, we find that five residues have a cutoff value above −0.9 kcal/mol, corresponding to a weak hydrogen bond, with values ranging from −0.91 kcal/mol for residues Phe127 and Thr189 to −2.1 for residue Leu179, as shown in Figure 7.

### 2.6. Giardia lamblia Viability and Adherence Assays

Based on our in silico results, we assayed the effects of DB07352, DB08399, and DB15584 on the viability of *G. lamblia* trophozoites of a clinical isolate resistant to nitazoxanide. Since previous reports show the effects of pomegranate peel extract on the viability and adherence of *G. lamblia* trophozoites, assays with DB15584 helped us for comparison. 

**Figure 8:**
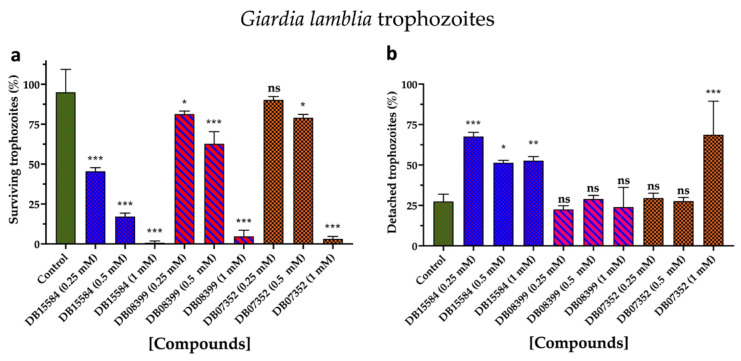
Effects on viability and adherence of *G. lamblia* trophozoites. Dose-response effects on viability of trophozoites with DB15584, DB08399, and DB07352 (**a**). Effects on trophozoites adherence of DB15584, DB08399, and DB07352 (**b**). ns: not significant, * *p* < 0.05, ** *p* < 0.01, *** *p* < 0.001.

As for DB15584, the DB07352 and DB08399 showed significant dose–response effects in terms of the viability of *G. lamblia* trophozoites, with DB08399 being the most effective of our two proposed compounds (Figure 8a). The effect observed on trophozoite attachment was not significant with DB07352 and DB08399 except for the highest concentration used of DB07352 (Figure 8b). Conversely, by observing its effects on adherence, DB15584 reinforced its mechanism of action by disturbing the expression or function of cytoskeletal proteins but did not discard the possibility of targeting other proteins such as *Gl*TBP. On the other hand, the whole analysis of these assays supports the idea of functional protein targets for DB07352 and DB08399 (likely *Gl*TBP) rather than structural proteins, as occurs for DB15584.

## 3. Discussion

Although transcription factors have been considered an undruggable target, recent evidence points to them as promising therapeutic targets for the treatment of various diseases such as cancer, and their potential use in the treatment of parasitic diseases has even been suggested [19,22]. Even though the TBP is an essential element for transcription and almost its entire surface interacts with other transcription factors or DNA, the use of the TBP as a therapeutic target has been under research [15]. There are two precedents in which TBP has been identified as a target. The first occurs indirectly through the inhibition of kinases responsible for phosphorylating the TBP and thereby modifying its affinity for the gene promoter region [41,42]. The other case comprises multiple examples, especially in the field of cancer, where alkylating agents that bind to DNA hinder the proper assembly of the TBP on the gene promoter region, interacting with the concave region of the TBP’s characteristic saddle-shaped structure [22]. It is worth mentioning that recent studies conducted by Santiago et al. [15] identify several regions of the convex part of the TBP protein in protists, such as *E. histolytica* and *P. falciparum*, as susceptible therapeutic targets due to the morphological and amino acid sequence differences they exhibit. It has been postulated that inhibiting protein–protein or protein–DNA interactions using small molecules could cause a disruption in transcription that is detrimental to the parasite [19,22]. Therefore, TBPs with sufficient sequence divergence are susceptible to being used as a therapeutic target. An example of this is the TBP of *T. vaginalis*, which exhibits such divergence that it is not functional in other organisms like *S. cerevisiae*, as determined through complementation assays [43]. Our analysis reveals that the NC2 motif of the TBP in the parasite studied here is highly divergent in sequence, as no amino acid residue is conserved in *Gl*TBP compared to the *Hs*TBP (Figure 1). Additionally, a morphological change in the cavity is evident, which could potentially accommodate small molecules that hinder the proper assembly of the pre-initiation complex without affecting *Hs*TBP, similar to what Santiago et al. propose for the TBP of *E. histolytica* and *P. falciparum* [15].

The main reasons why transcription factors are considered undruggable are the absence of defined binding pockets, which typically serve as the target site for small-molecule drugs. Unlike other proteins that have active sites, transcription factors interact with DNA and other proteins over large, flat surfaces, making it difficult to design molecules that can effectively and specifically bind to these interfaces. Additionally, transcription factors often involve complex protein–protein interactions within multiprotein complexes. Disrupting these interactions without affecting other cellular processes presents a significant challenge in drug design [44].

In the context of drug discovery and design, recent advances are beginning to address these challenges. For example, the development of proteolysis-targeting chimeras (PROTACs) has shown promise in targeting transcription factors by promoting their degradation instead of inhibition. Furthermore, advancements in structural biology and computational modeling are providing new insights into the dynamic conformations of transcription factors, potentially revealing novel druggable sites. Our research proposes the NC2 motif as a potential therapeutic target for small-molecule drugs, which can competitively inhibit the assembly of the transcription preinitiation complex (PIC) in this parasite specifically, significantly impairing its viability [44].

In this research, we assessed over 11,000 compounds sourced from Drug Bank with the objective of identifying potential candidates for inhibiting the TBP in this medically significant protist.

In *Gl*TBP, molecular docking and molecular dynamics analyses revealed that the compound DB07352 (Apigenin) stood out, showing a binding energy of −7.459 kcal/mol and a selectivity of −1.806 compared to its human homolog. It is important to note that previous reports have indicated the anti-*Giardia* effect of phenolic and flavonoid compounds found in oregano extract, among which DB07352 is included [34]. Furthermore, molecular dynamics simulations suggest a stable binding of this compound to *Gl*TBP, with no disruptions in the protein’s structure throughout the simulation. Furthermore, DB07352 exhibited three hydrophobic interactions (with F127, F144, and K178) and formed four hydrogen bonds with S128, S129, K145, and K178. Similarly, compound DB08399 (Piceatannol) displayed a binding energy of −7.318 kcal/mol and a selectivity of −2.06. Although its anti-*Giardia* activity has been previously reported as acting on microtubule assembly [26], its inhibitory potential against *Gl*TBP is a new perspective supported by our molecular dynamics simulations. Lastly, this compound displayed three hydrophobic interactions with F127, F144, and K178 and formed four hydrogen bonds with S128, S129, K145, and E181. In summary, the predominant reported interactions are hydrophobic interactions and hydrogen bonds. It is noteworthy that these types of interactions are considered crucial for the structure and stability of protein–ligand complexes. Specifically, F127, K178, and F144 are present in 100%, 90%, and 80% of cases, respectively, forming hydrophobic interactions with some of the top ten compounds with better selectivity. Additionally, S129, S128, and L145 interact with any compound in 90%, 80%, and 80%, respectively, establishing hydrogen bond interactions. According to our MM-PBSA analysis, the residues that contribute the most energy to the binding of the DB07352 complex are L179 with −2.1 kcal/mol; I126; and L183 with −1.2 kcal/mol, while for DB08399, they are Y186 and L183. 

Also, DB07352 and DB08399 showed dose-dependent anti-*Giardia* activity. Since the detaching process of trophozoites was not significant by assaying these two compounds, their mechanism of action is likely more related to targeting functional rather than structural proteins. Based on the latter, further studies to demonstrate the inhibitory properties of these compounds against *Gl*TBP seem promising.

Additionally, of the top ten compounds with the best selectivity index for *Gl*TBP compared to *Hs*TBP, amino acid residue K178 stands out, forming nine hydrophobic interactions and five hydrogen bonds with various compounds. They are followed by F127, for which ten hydrophobic interactions are reported, along with a π-stacking interaction. Subsequently, residues S129 and K145 each present nine interactions, mainly in the form of hydrogen bonds, while residues S128 and E181 show eight and six interactions, respectively, primarily in the form of hydrogen bonds. It is worth noting that all these amino acid residues are classified as part of the NC2 motif in our structural alignment, except for F127 (see Figure 1).

## 4. Materials and Methods

### 4.1. Protein Sequence Retrieval

The protein sequence of *Gl*TBP was obtained from GiardiaDB (http://GiardiaDB.org, accessed on 2 August 2023), while the sequences of *T. cruzi* (*Tc*TBP) and *L. mexicana* (*Lm*TBP) were retrieved from TriTrypDB (https://tritrypdb.org, accessed on 2 August 2023). On the other hand, the RCSB PDB database was used to search for the resolved structures of eukaryotic TBPs, this with the objective of obtaining a structural alignment, which allows us to delimit the carboxyl-terminal domain of the sequences to be evaluated, leading to the retrieval of the TBPs’ structures from *Homo sapiens* (*Hs*TBP; PDB ID: 1JFI), *A. thaliana* (PDB ID: 1QNA), *S. cerevisiae* (PDB ID: 1NH2), and *E. cuniculi* (PDB ID: 3OCI). Additionally, the protein sequences of other eukaryotes were retrieved from NCBI using the BlastP tool (https://blast.ncbi.nlm.nih.gov/Blast.cgi, accessed on 2 August 2023) using the 1JFI sequence as a query sequence. The additional protein sequences belong to *E. histolytica* (XP_654935), *D. melanogaster* (NP_523805), and *T. flavidus* (XP_056871520). It is worth mentioning that these last sequences were retrieved to strengthen our multiple sequence alignment and to evaluate the conservation of amino acid residues in the NC2 motif.

### 4.2. Multiple Sequence Alignments

A structural alignment was performed with the PDBeFold server (https://www.ebi.ac.uk/msd-srv/ssm/, accessed on 5 August 2023) of the Protein Data Bank Europe (PDBe) with the four structures retrieved from the RCSB PDB (1JFI, 1QNA, 1NH2, and 3OCI). Subsequently, this alignment was taken as a basis to align the rest of the sequences retrieved from the specialized database (GiardiaDB) and those from the NCBI database. Based on the previous structural alignment, the second alignment was performed with the Clustal omega algorithm. Once this last alignment was obtained, the protein sequences of all those organisms whose TBP have not been resolved experimentally were delimited for the DNA-binding domain (C-terminal) to later predict their three-dimensional structure using the AlphaFold2 server [45,46]. Once all the models were obtained, the amino acids involved in recognizing the Negative Cofactor 2 (NC2) were highlighted, both in experimental (crystallographic structures) and in silico models (3D predicted models), based on the 1JFI structure, which is the only structure in the RCSB PDB database of the TBP in complex with the general transcription factor NC2.

### 4.3. Determination of Ligand Binding Site

Several methods were used to predict the possible binding site of GlTBP; the DoGSiteScorer Binding site server was used [47]. Additionally, blind docking was performed between GlTBP and the 7969 compounds to be evaluated [48,49], and a bibliographic search determined that the NC2 binding site is a potential site susceptible to inhibition [15,16]. 

### 4.4. Molecular Docking

Protein preparation. *Hs*TBP (PDB ID: 1JFI) was prepared for molecular docking using UCSF Chimera software (v.1.16) [50]. The ligands and water molecules were removed from the PDB: 1JFI file, which corresponds to the *Homo sapiens* TBP taken from the RCSB PDB database. Subsequently, the 3D predicted model of *G. lamblia* and the 1JFI crystallographic structure, which was previously prepared, were protonated, and charges were added (Gasteiger field force) using AutoDock Tools software v.4.2 [51]. Finally, the two files were saved in pdbqt format.

Ligand preparation. We obtained a set of 11,584 DrugBank (https://go.drugbank.com/, accessed on 10 August 2023) compounds representing FDA-approved drugs in sdf format. Afterward, each compound was converted to mol format using OpenBabel v.2.4.1 and filtered based on Lipinski’s Rule of Five, which includes pH 7.0 [52]. After filtering the compounds, a total of 7969 compounds were selected. These compounds were then prepared using MGLtools software (version 1.5.6.), which involved processes such as minimization, the addition of polar hydrogens, and conversion to pdbqt format. 

Determination of grid for molecular docking. The three-dimensional grid was defined using the 14 amino acid residues that interact with NC2, as identified from the resolved crystallographic structure of the TBP in complex with NC2 (PDB ID: 1JFI). The positioning of these residues in the parasite sequence was determined through the structural alignment. The grid for *Gl*TBP had the following coordinates and size: the X, Y, and Z coordinates were −7.421, 6.858, and 19.068, respectively, with a size of 32 Angstroms in each of the three dimensions.

Subsequently, molecular docking analysis was conducted using AutoDock Vina [53,54], with *Gl*TBPs and *Hs*TBP as the receptors, against the 7969 compounds previously prepared. Next, the binding free energy (BFE) of each compound in *Hs*TBP was compared with their respective values in *Gl*TBP to identify the 10 compounds that showed greater selectivity toward the *Gl*TBP, as indicated by the lowest BFE. Finally, the interactions of the complexes belonging to the compounds with the best selectivity were obtained using the PLIP computational tool (v. 2.2.2) [55]. 

### 4.5. Molecular Dynamics 

Molecular dynamics simulation was carried out by triplicate using the GROMACS v. 2018.4 software suite [56]. Before starting the simulation, the necessary topology and parameter files were generated using the ACPYPE (AnteChamber PYthon Parser interfacE) software (v. 2022.1.3) with the General Amber Force Field (GAFF) for the compounds of interest [57].

In the initial stage, we initiated system solvation by introducing water molecules into a dodecahedral model, ensuring a minimum distance of 10 angstroms between the water molecules and the dodecahedron’s walls. We employed the TIP3P water model for solvation. 

In the second phase, we introduced ions (cations or anions) into the system to neutralize charges, thereby mimicking physiological cellular conditions. Following this, we performed system minimization using the steepest descent algorithm, involving a total of 50,000 steps.

In the third stage, equilibration was conducted under NVT conditions (constant number of particles, volume, and temperature). A V-rescale thermostat with a time constant of 0.1 picoseconds was employed for temperature coupling, and initial velocities were assigned to the particles following a Maxwellian distribution.

In the fourth stage, equilibration was carried out under NPT conditions (constant number of particles, volume, temperature, and pressure). A V-rescale thermostat with a time constant of 0.1 picoseconds was employed for temperature coupling. Pressure coupling was achieved using the Berendsen method with a time constant of 2.0 picoseconds. In both conditions, a reference temperature of 300 K was maintained for 100 picoseconds.

After the equilibration stage, the simulation was conducted with a trajectory of 120 nanoseconds. Temperature coupling was maintained using a V-rescale thermostat with a time constant of 0.1 picoseconds, while pressure coupling was achieved using the Parrinello–Rahman method with a time constant of 2.0 picoseconds, keeping a reference temperature of 300 K. Finally, the stability of the complexes was assessed by calculating the values of RMSD (Root-Mean-Square Deviation), RMSF (Root-Mean-Square Fluctuation), and the gyration radius.

### 4.6. MM-PBSA Analysis

The MM-PBSA analysis was carried out using the last 40 ns of the molecular dynamics simulation. The ∆G_b_ values for the two compounds with inhibitory potential against *Gl*TBP were calculated using the g_mmpbsa program (v. 5.1.2) [58]. Additionally, the residues involved in the interactions, along with their partial contribution to the binding free energy, were recovered using the MmPb-SaDecomp.py script.

### 4.7. Susceptibility Assays and Parasites

DB15584 was dissolved in ethanol to obtain a 50 mM solution, whereas DB08399 and DB07352 were dissolved in dimethyl sulfoxide (DMSO) to obtain a 50 mM solution for each one. Drugs were diluted in *G. lamblia* culture medium to obtain the working concentrations of 0.25, 0.5, and 1 mM.

For susceptibility assays, the clinical isolate N1-INP of *G. lamblia* of the assemblage AI was used [59]. Parasites were cultured in TYI-S-33 modified medium supplemented with 10% calf serum and bovine bilis. Trophozoites (5 × 10^4^/mL) were incubated with the drugs at different concentrations (0.25, 0.5, and 1 mM) for 24 h at 37 °C. Supernatants were withdrawn in new tubes after incubation to analyze the cell detaching process. Supernatants and samples with attached trophozoites were chilled on ice for 20 min, centrifuged at 380× *g* for 15 min at 4 °C, and resuspended in phosphate-buffered saline (PBS). Trophozoite samples were incubated in trypan blue solution (1:1) for 5 min and counted with a hemocytometer. Trophozoites with DMSO or ethanol (at the highest concentration used in working solutions) were included as negative controls. Three independent assays were performed in triplicate per compound concentration.

## 5. Conclusions

In summary, this study has identified several compounds with potential inhibitory activity against the *Gl*TBPs, which may have significant implications for the development of targeted therapies against giardiasis. The low conservation of amino acids in the active site favors the development and research of specific therapies for these transcription factors, which are essential for the parasite’s survival without adverse effects on the host. The results obtained from compounds DB07352 and DB08399 via molecular docking, molecular dynamics simulations, and trophozoite susceptibility provide a solid foundation for future research.

## Figures and Tables

**Table 1:** Top ten compounds with the best binding free energy (BFE) and interaction profile (IP) against *Gl*TBP.

Compound	Target Reported	Giardicidal Effect	BFE and IP	Other Functions
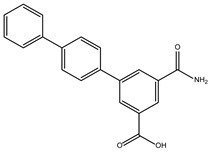 DB04583	Dihydroorotate dehydrogenase (quinone), mitochondrial	Negative	−8.365 kcal/mol. **HI.** I126, F127, F144, K178, E181; **HB.** S128, S129, K145; SB. K178; π-stacking. F127.	New inhibitor of Human dihydroorotate dehydrogenase [25].
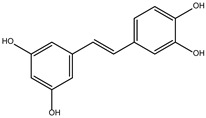 DB08399 (Piceatannol)	ATP synthase subunit (alpha, beta, gamma) mitochondrial.	Positive	−7.318 kcal/mol. **HI.** F127, F144, K178; **HB.** S128, S129, K145, E181.	Effect on the assembly of microtubules [26]. Piceatannol blocks both the hydrolysis and synthesis of ATP through the ATP synthase mitochondrial [27].
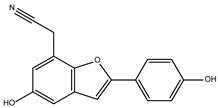 DB06927	Estrogen receptor beta. Nuclear receptor coactivator 1. Estrogen receptor alpha.	Negative	−7.749 kcal/mol. **HI.** F127, F144, K178; **HB.** S128, S129, K145, E181.	Not available.
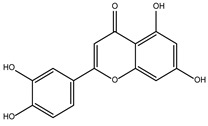 DB15584 (Luteoline)	Not Available.	Positive	−7.720 kcal/mol. **HI.** F127, K178; **HB.** S128, S129, K145, K178, G182.	Effect on the growth and decrease in the adhesion of trophozoites treated with 200 μg/mL. IC_50_ of 179 μg/mL [28]. Luteolin is a flavonoid with anti-inflammatory actions that bind PPARγ [29].
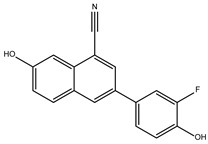 DB06875 (ERB-196)	Estrogen receptor beta. Nuclear receptor coactivator 1.	Negative	−8.090 kcal/mol. **HI.** F127, F144, K178; **HB.** V143, K145, K178, E181.	WAY-202196 (DB06875) was effective in two inflammation models, suggesting that targeting estrogen receptors may be medically helpful in treating certain chronic inflammatory diseases [30].
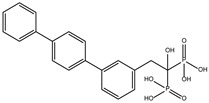 DB07409	Ditrans-polycis-undecaprenyl-diphosphate synthase ((2E,6E)-farnesyl-diphosphate specific).	Negative	−7.888 kcal/mol. **HI.** I126, F127, F144, K178, E181; **HB.** I126, S128, S129, V143, K145.	Inhibitor of undecaprenyl diphosphate synthase [31].
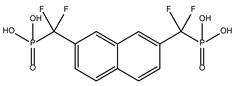 DB03154	Tyrosine-protein phosphatase non-receptor type 1.	Negative	−7.712 kcal/mol. **HI.** F127, F144, K178; **HB.** S128, S129; **HalB.** E181.	Inhibitor of Human Tyrosine Phosphatase 1B [32]. This enzyme is an attractive therapeutic target because it regulates insulin sensitivity [32].
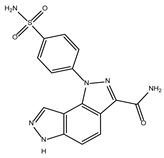 DB08527	Cyclin-A2. Cyclin-dependent kinase 2.	Negative	−7.743 kcal/mol. **HI.** I126, F127, F144, K178; **HB.** S128, S129, K145, K178.	A new class of CDK2 inhibitors [33]. Anti-proliferative activity [33].
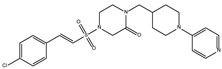 DB08745	Coagulation factor X.	Negative	−7.355 kcal/mol. **HI.** I126, F127, V143, K145; **HB.** S129, N150.	Not available.
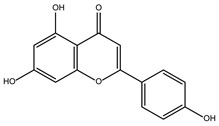 DB07352 (Apigenin)	3-hydroxyacyl-[acyl-carrier-protein] dehydratase FabZ.	Positive	−7.459 kcal/mol. **HI.** F127, F144, K178; **HB.** S128, S129, K145, K178.	Anti-giardia activity is due to the presence of phenolic compounds and flavonoids, among which is Apigenin [34]. tankyrase inhibitor [35]. This compound has been proposed as effective drugs in the therapy of TTR amyloidosis [36]. DAPK1 (inhibitor) is a possible target for treating acute ischemic stroke and endometrial adenocarcinomas [37]. Competitive inhibitor against protein dehydratase from Helicobacter pylori (HpFabZ) [38]. This compound may act as Transthyretin (TTR) amyloid inhibitor [39].

BFE: Binding free energy; IP: Interaction profile; HI: Hydrophobic interactions; HB: Hydrogen bonds.

**Table 2:** Values of molecular interactions and binding energy in TBP/ligand complexes.

TBP/Ligand	∆E_vdw_	∆E_ele_	∆G_polar_	∆G_SA_	∆G_b_
*Gl*TBP/DB07352	−29.58 ± 0.17	−1.64 ± 0.12	15.25 ± 0.16	−3.31 ± 0.01	−19.28 ± 0.15
*Gl*TBP/DB08399	−23.53 ± 0.19	−9.32 ± 0.17	21.89 ± 0.18	−3.21 ± 0.01	−14.16 ± 0.17

## Data Availability

All data are available online. No unpublished data have been used in this paper.
